# The Dioxin Crisis as Experiment To Determine Poultry-Related *Campylobacter* Enteritis

**DOI:** 10.3201/eid0801.010129

**Published:** 2002-01

**Authors:** Akke Vellinga, Frank Van Loock

**Affiliations:** Institute of Public Health–Louis Pasteur, Brussels, Belgium

**Keywords:** Campylobacter, poultry, dioxin crisis, Belgium, infection, ecological study

## Abstract

In June 1999, the dioxin crisis, caused by dioxin-contaminated feed components, exploded in Belgium, resulting in withdrawal of chicken and eggs from the market. Through the sentinel surveillance system, a decrease in *Campylobacter* infections during June 1999 was noticed. A model was generated with the reports from preceding years (1994 to 1998), and a prediction of the number of infections in 1999 was calculated. The model shows a significant decline (40%) in the number of infections, mainly because of the withdrawal of poultry. The use of a disaster as an epidemiologic tool offers a unique opportunity to observe exceptional changes in the occurrence of infections or other diseases.

In 1999, Belgium had a dioxin crisis caused by dioxin-contaminated feed being fed to livestock (*1*,*2*). The problem started at the end of January, when contaminated feed was processed; however, official notification of the crisis did not occur until the end of May (*3*). The source of the contamination was a fat-rendering company, where transformer oil with high levels of polychlorinated biphenyls (PCBs) and dioxins was used to manufacture animal foods. On May 28 (week 21), Belgian authorities ordered the withdrawal from sale of Belgian poultry and eggs; other European countries and Russia followed suit. On June 2, the European Community widened the ban and ordered the destruction of all food items containing >2% egg product and food containing chicken produced from January 15 to June 1 from infected farms (*3*). On June 4, 1999, the Belgian government issued a commerce embargo of meat products (pork and beef) with a minimum of 25% fat content, not applicable for dairy products. Meat was not withdrawn from sale.

In Belgium, a surveillance system of sentinel (n=127) and reference (n=38) laboratories, set up in 1983, reports on a voluntary basis on a list of organisms, including *Campylobacter*. Of all recognized private and hospital laboratories, 46% contribute to the surveillance system, which covers 35 of Belgian's 43 districts (*4*).

The number of registered *Campylobacter* infections increased from 2,534 cases in 1985 to 6,610 cases in 1998 and 6,521 in 1999. In Belgium, *Campylobacter* enteritis (campylobacteriosis) is mainly caused by *Campylobacter jejuni* (80% of the isolates) and *C. coli* (12%) (*4*).

Campylobacteriosis is a common form of infective diarrhea in industrialized countries; most infections are sporadic, and 80% are believed to be foodborne (*5*,*6*). *Campylobacter* can be isolated from many species of wild and domesticated animals, which are mainly asymptomatic carriers. Of farm animals, poultry and pigs are most frequently infected (*5*,*7*). Various risk factors have been suggested on the basis of case-control studies and outbreak investigations, including handling chicken (*8*–*10*); eating not fully cooked chicken (*11*–*13*); eating commercially prepared chicken (*13*,*14*), sausages (*10*), and barbecue (*10*,*15*,*16*); exposure to farm or domesticated animals (*10*–*13*); or consumption of raw milk (*12*,*13*,*16*).

The dioxin crisis had implications for public health on more levels than the direct health effects of dioxin (*17*). Whereas the level of dioxin contamination and the health outcomes require research projects over long periods, one of the direct effects of this crisis could be noticed in the number of *Campylobacter* infections. This unique event made it possible to investigate the withdrawal of particular food products from the market and their contribution to campylobacteriosis.

## Methods

**Figure 1:**
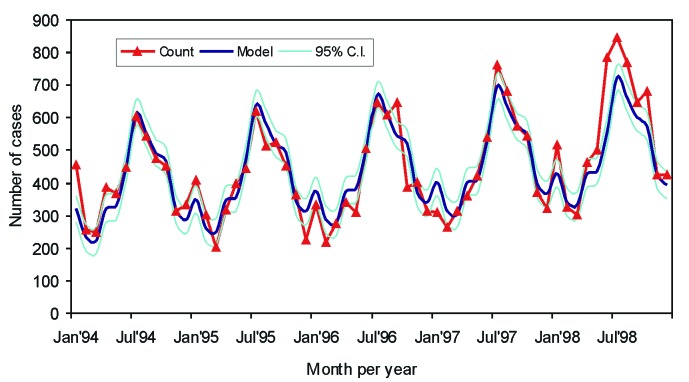
Campylobacteriosis in Belgium from 1994 through 1998, fit of the model.

To estimate the effect of the dioxin crisis on the number of *Campylobacter* infections, a model was designed by which the number of expected cases for 1999 could be calculated. This model was based on the data collected by the sentinel laboratory surveillance network from 1994 to 1998. The cumulative numbers per week were used to drive a monthly chronologic series for 1994 to 1998 and modeled according to the Fourier transformation (*18*,*19*). This procedure explains the cyclic patterns of data by spectral analysis; a complex time series with cyclic components is decomposed into sine and cosine terms describing the seasonal changes and a linear term to identify the trend (Figure 1).

The final model has three cyclical terms (52, 26, and 13 weeks), i.e., a strong yearly variation and two harmonics at the half and quarter year.

**Figure 2:**
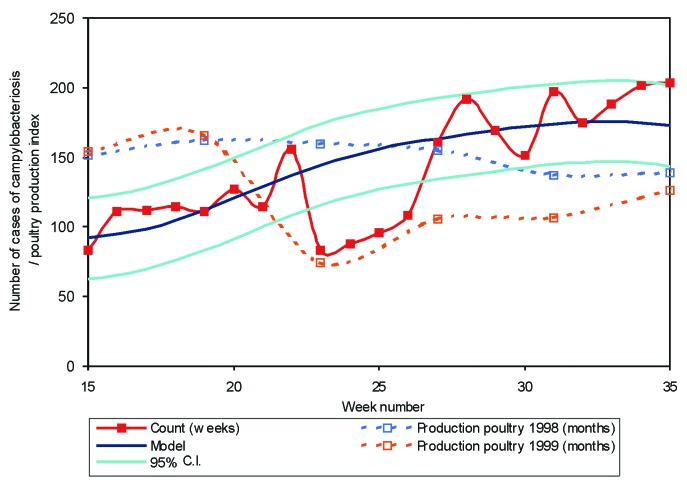
Campylobacteriosis in Belgium at period of dioxin crisis, model 1994-1998 and poultry production index.

The linear trend shows a yearly increase from 1994 to 1998, with a slope of 15%. When the cyclical contributions are included, r²=0,86, that is, 86% of the variation in the number of the *Campylobacter* infections can be explained by the model. The epidemic threshold is set at a distance of 1.96 standard deviations (SD)(95% confidence interval [CI]), which has shown to be useful for distinguishing epidemic increases from random variation (*18*), or (as for the dioxin crisis) exceptional, epidemic decrease. Outbreaks over the years were smoothed for this calculation. Figure 2 shows the model, including the 95% CI and the actual numbers by which it was calculated. Poultry production per workday for 1998 and 1999 is also shown, representing the production per workday in indices with 1995 as reference year (1995 = 100) (*20*).

Age and gender distribution during the crisis was compared with the monthly distribution of age and gender in previous years. The model was made with MS Excel 2000; SPSS version 9.0 was used to compare distributions.

## Results

Figure 2 shows the model, including the 95% CI as calculated from the numbers in the previous years during a period of 20 weeks (middle of April to the end of August, weeks 15 to 35) as well as the actual count for this period in 1999. The number of *Campylobacter* infections analyzed by the sentinel laboratory surveillance network fit the 95% CI except during the dioxin crisis (from week 21), when all poultry and eggs were taken off the supermarket shelves. The number of *Campylobacter* infections from week 23 to week 26 in 1999 is on average 94 cases per week or almost 40% lower than the expected average of 153 cases per week (SD_model_=15 cases/week; SD = standard deviation). Overall, for the month of June (week 23 to 26), the expected number of infections was 643 (ST_model_=61 cases/month) while the actual number of cases in 1999 was 375. The monthly calculated numbers follow the same trend as *Campylobacter* infections in 1999 and a similar decrease in numbers during the dioxin crisis. After 4 weeks, the ban was lifted, and the number of *Campylobacter* infections returned to the interval calculated by the model. Poultry production, even though over a longer period, also returned to levels comparable with the month of the previous year (1998). There was no difference in age or gender distribution of *Campylobacter* infections during the crisis compared with the rest of the year.

## Discussion

The impact of the dioxin crisis can be detected in various disciplines. The economic impact is probably the easiest to determine, as this aspect is data driven. However, the dioxin crisis had a tremendous impact on health and health-related matters, including food consumption. The sudden change in food consumption, related to the withdrawal of poultry and eggs, had an immediate effect on the number of foodborne diseases, e.g., *Campylobacter*. The fitted model shows an unexpected decline in June 1999. Even though the decline in numbers is not exceptional, the moment at which this happens is. Looking at other declines in the number of *Campylobacter* infections, as in February 1996 or February 1997, these happen during a downward trend in numbers, while the drop in June 1999 occurs when numbers would normally be increasing.

The decline in campylobacteriosis and the lack of poultry in shops lasted 4 weeks, exactly the period from seizing all Belgian chicken and egg products from the supermarket shelves until the return of these items. This supports a direct link and contradicts a possible “ecological fallacy” as the time frame, which has an abrupt beginning and end, is similar for both the dioxin crisis and the decline in number of *Campylobacter* infections (*21*). During this period no other events possibly explaining the decline in numbers are known to have occurred. A major concern with ecologic studies is often the flue line (generally, the geographic boundaries of the occurrence of the risk factor and the occurrence of the illness), as was the case in the European study of the association between olive oil and cancer (*22*). The dioxin crisis differs from the commonly analyzed ecologic studies in this geographic aspect, since the borders of the impact of the dioxin crisis are the same as the borders of a well-established surveillance system.

*Campylobacter* is associated with several risk factors and risk behaviors, such as contact with farm and domesticated animals (mainly cats and kittens) (*10*–*12*) or recent history of antibiotic use (*14*). However, the dioxin crisis would not have an immediate effect on these factors, as it was primarily a food scare. Drinking raw milk, occasionally found to be a risk factor (*12*,*16*), is also unlikely to have a contributed to the decline in numbers since milk products were not taken off the shelves.

Meat, in particular pork prepared on the barbecue, is generally accepted to be an important risk factor (*10*,*16*). Pork meat is associated with *C. coli*, which accounted for 12% (in 1998) and 21% (in 1999) of all *Campylobacter* infections (*4*). Meat remained available during the crisis, which might explain the small increase in *C.* *coli* infections, as it is imaginable that chicken, which is the main source of *C. jejuni* infections, was replaced by pork during the crisis.

Eggs were not on sale during the crisis, but they are generally not associated with *Campylobacter* contamination. (A possible link of eggs with *Salmonella* infections will be examined.)

The withdrawal of chicken and all related products from the supermarket during the dioxin crisis is the most likely reason for the sudden decline in *Campylobacter* infections. Chicken is found to be the principal source of infection in most case-control studies (*11*,*13*,*14*,*16*). In Seattle, rare, raw, and cooked chicken were all significantly associated with *Campylobacter* infection (*8*). Undercooked chicken was found to be a risk factor in a Colorado study (*12*). Handling raw and even frozen chicken, possibly because of cross-contamination in the kitchen, has also been significantly associated with campylobacteriosis (*10*). As all Belgian poultry was withdrawn, our data allow an estimation of the number of *Campylobacter* infections directly related to poultry.

The decline in the number of *Campylobacter* infections in Belgium by 40% was due to the withdrawal of Belgian poultry from the market. In 1999, 199,251 tons of poultry meat was available for human consumption; 81,261 tons (41%) was imported (*23*). Foreign poultry remained available on the market.

According to a marketing bureau that investigates trends in shopping behavior of 3,000 families in Belgium, eating habits have changed little because of the dioxin crisis (*24*); moreover, the overall purchase of poultry in 1999 increased by almost 9%. However, a shift was seen in the quality of the poultry sold; after the crisis, consumers preferred chicken with some sort of quality label, even though the current labels do not specifically address contamination issues.

Besides the 40% decrease in *Campylobacter* infections during the dioxin crisis, this experiment also highlights the remaining baseline of 60%, averaging 75 infections a month. According to an analysis of foodborne disease information in the United States (1999), only 80% of the *Campylobacter* spp. infections are estimated to be foodborne (*5*). Furthermore, as only Belgian chicken was banned and non-Belgian poultry was still on sale, a number of poultry-related infections are still present in the reported numbers. With at least 40% of the *Campylobacter* infections in Belgium explained by poultry and 20% by non-foodborne causes, the source of the remaining infections should be further explored and investigated.

The use of a disaster as an epidemiologic tool offers a unique opportunity to observe exceptional changes in the occurrence of infections or other diseases. The causes or consequences of the crisis can serve as treatment in an uncontrolled natural experiment. The dioxin crisis as experiment showed that >40% of the *Campylobacter* infections can be attributed to poultry.
